# Misaligned by design: evaluating and deploying generative AI for the real-world conditions of primary care: a Nordic and European perspective

**DOI:** 10.1080/02813432.2026.2735209

**Published:** 2026-09-19

**Authors:** Florian O. Stummer, Timothy Tsai, Carl Wikberg, Veronica Milos Nymberg, Ilja Radlgruber, Angelina Müller, M. Riegler, Ferdinando Petrazzuoli, Shlomo Vinker, Thomas Frese

**Affiliations:** a Institute of General Practice and Family Medicine, Martin-Luther-Universität Halle-Wittenberg, Halle (Saale), Germany; b Stanford Healthcare AI Applied Research Team, Division of Primary Care and Population Health, Stanford University School of Medicine, Stanford, CA, USA; c General Practice/Family Medicine, School of Public Health and Community Medicine, Sahlgrenska Academy, University of Gothenburg, Gothenburg, Sweden; d Research, Education, Development & Innovation (RED-I), Primary Health Care, Region Västra Götaland, Gothenburg, Sweden; e Center for Primary Health Care Research, Department of Clinical Sciences Malmö, Lund University, Malmö, Sweden; f University Clinic Primary Care, Skåne University Hospital, Malmö, Sweden; g Faculty of Medicine, Sigmund Freud Private University, Vienna, Austria; h Institute of General Practice, Goethe-University, Frankfurt am Main, Germany; i Simula Research Laboratory, Simula Cyber Security, Oslo, Norway; j European Rural and Isolated Practitioners Association (EURIPA), Pordenone, Italy; k Department of Family Medicine, Sackler Faculty of Medicine, Tel Aviv University, Tel Aviv, Israel; l Leumit Health Services, Tel Aviv, Israel

**Keywords:** Generative AI, large language models, primary care, general practice, Nordic primary care, benchmarks, European Health Data Space, EU AI act, clinical decision support, trust

## Abstract

**Background:**

Large language models (LLMs) have entered clinical and consumer use faster than primary-care researchers can evaluate them, and the dominant benchmarks and deployment strategies were shaped in hospital settings rather than primary care. The evidence is mixed: GPT-4 scored below general practitioners on the Swedish family-medicine specialist examination, while Brodeur et al. (2026) found an LLM outscoring the several hundred physicians who worked the same hospital-based cases. The decisive problem is therefore not raw performance but misalignment between how these systems are evaluated and deployed and real primary-care conditions.

**Main body:**

We set out six such conditions: clinical, relational, organisational, cultural, linguistic, and epistemic, and show where current development and evaluation misalign with each. We then state what must change: benchmarks built for multimorbidity, longitudinal reasoning, and continuity; correction of cultural, linguistic, and socio-economic underrepresentation in training corpora, with Nordic registers as counterweight; outputs tied to a verifiable source and assessed independently, not vendor-certified; generative AI (GenAI) deployed as a tool under the Five Rights; and a European and Nordic agenda anchored in the European Health Data Space and aligned with the EU AI Act. We give a success criterion for each recommendation, and separate what is specific to primary care from general clinical-AI principles.

**Conclusions:**

Accuracy alone will not make these systems trustworthy in primary care, and some proposed uses may not survive evaluation under real-world conditions. Whether GenAI earns a place here is an empirical question the Nordic primary-care research community is well placed to answer.

## Background

In November 2022, OpenAI released the large language model (LLM) GPT-3.5 to the world. For primary care, the technology arrived as both a potential relief from administrative overload and a workforce in decline, and as a new ethical and operational challenge to integrate sustainably [1–4]. As the initial point of contact for most healthcare encounters, primary care is the central axis of the system. It requires comprehensive, longitudinal, and person-centered care, supported by technologies tailored to real-world heterogeneity and communication complexity. The question that decides whether these systems belong in primary care is not how accurate they can be made, but whether they can be trusted by the people who must use and live with them.

Trust is the organising concept of this paper, and it is not one thing. It operates on at least three levels, each calling for different safeguards: institutional trust in governance, certification, and independent assurance; clinician trust in the reliability and verifiability of a specific tool at the point of care; and patient trust in care that is mediated, in part, by a machine. A system can be technically accurate and still fail on any of these. Throughout this paper we treat trust as this layered construct rather than as a synonym for accuracy, and we return to it when we set out what successful implementation would look like.

The evidence on capability is mixed, and it matters what kind of capability is meant. We use capability for measured performance on clinical tasks set for a model, which is not the same as fitness for use in practice. Almost all of that evidence concerns diagnostic accuracy and case-based reasoning on written vignettes; far less concerns communication, longitudinal management, or documentation quality, and almost none concerns performance sustained across repeated encounters with the same patient. On the first of these, an observational study using the Swedish family-medicine specialist examination found that, on complex primary-care cases, GPT-4 scored on average 1.6 points lower than randomly selected human responses and 2.7 points lower than top-tier human responses on a 10-point scale [5]; a rerun after the release of GPT-4o narrowed the gap to the randomly selected human responses to 0.7 points, which is itself a reminder of how quickly the ground moves. More recent work points the other way: Brodeur et al. report an LLM matching or exceeding the reference scores of the several hundred physicians who worked the same cases, across five reasoning experiments and a real-world emergency-department arm [6]. That evidence was generated in hospital care. We use hospital-based care for both secondary (general hospital and emergency) and tertiary (specialised referral) settings, because what matters here is what unites them: a case-mix already selected and triaged, with higher disease prevalence and narrower differential fields. The relevant contrast is therefore hospital-based versus primary care rather than tertiary versus secondary, and Brodeur et al. sit on the hospital side of it; the accompanying commentary itself calls for prospective real-world trials [7]. The lesson is not that GenAI is too weak for primary care, but that benchmark capability and primary-care fitness are not the same thing.

Nordic primary-care research carries a long tradition of grounded multidisciplinary, multi-method empirical work, strengthened by high-quality regional registries that have shaped a generation of comparative studies. This provides a rare and comprehensive empirical counterweight.

A terminological distinction is needed before proceeding. Traditional artificial intelligence (AI) typically operates on structured data to make deterministic predictions such as classification, triage, or risk scoring. LLMs fall under the category of generative AI (GenAI), capable of producing nuanced, context-sensitive language outputs. The distinction is critical: GenAI systems represent a new paradigm of interaction, synthesis, and decision support that remains largely unvalidated within primary care [8]. Failure to make this distinction risks conflating capabilities and misaligning expectations at the policy and clinical levels [9–15]. A second convention follows: we use primary care as the umbrella term for the setting throughout, and general practitioner (GP) for the clinician working in it, retaining general practice only where a cited source or a proper name uses it.

This paper argues that the development and evaluation of GenAI are misaligned with the real-world conditions of primary care, and that these conditions are clinical, relational, organisational, cultural, linguistic, and epistemic, not merely structural. We take each condition in turn, show where current practice misaligns with it, and then set out a European and Nordic research and implementation agenda, one informed not by GPs alone but by the interdisciplinary, multi-method community working in and with primary care, and workable for EU members (Sweden, Denmark, Finland), EEA members (Norway, Iceland), and the broader European primary-care community.

## Two distinct asks: administrative support and clinical decision support

Before asking how GenAI should be evaluated, it helps to be precise about what it is being asked to do, because two very different uses are routinely conflated.

The first is administrative: documentation, coding, and communication between colleagues and across sectors. The tools we refer to here are AI tools, specifically ambient documentation systems (AI scribes) that transcribe and structure the consultation, and LLM-based drafting of referrals and letters. These are already in regular use in Nordic primary care. Norwegian GPs using an AI scribe report saved time, reduced stress, and better consultation structure, alongside occasional transcription errors and hallucinations that make careful review necessary [16]. A Swedish real-world evaluation in an integrated provider covering primary, secondary, and hospital care, whose survey cohort worked mainly in general practice, reported self-reported documentation time per note falling from 6.69 to 4.72 min and lower administrative stress after introduction of an AI scribe [17]. This is where value is most readily demonstrated today. We call this work lower-stakes with one important qualification. It is lower-stakes only because the output is generated for a clinician who reviews and signs it, so an error is in principle visible and correctable at the point of entry, whereas an error in decision support acts on the reasoning itself and may never surface. Where review is perfunctory, or where the volume of generated text makes genuine review impractical, that assumption fails and a documentation error propagates through every subsequent encounter, referral, and coded record. The same Swedish evaluation is instructive on exactly this point: median editing time was 93 s and did not fall with continued use, and 138,196 of the 236,153 notes were excluded from the editing analysis because no editing time was recorded at all, leaving it open whether those notes were used as generated, edited elsewhere, or discarded [17]. Its design also illustrates the assurance gap this paper argues about: it is a single-arm retrospective evaluation reported partly by the vendor of the tool under study. The label therefore describes a design condition to be enforced, not a property of the task.

The second is clinical decision support, which is not a single task either. There is a real difference between supporting complex, whole-person generalist work, multimorbidity, psychosocial distress, the undifferentiated presentation, and supporting bounded, specific tasks such as screening risky polypharmacy for drug-drug and drug-disease interactions, or helping rule a rare diagnosis in or out. GenAI is least mature for the former and most immediately useful, in skilled hands, for the latter. Keeping these uses apart is itself a guard against misaligned expectations.

## Where current GenAI development and evaluation misalign with primary care

### Clinical

Primary care manages undifferentiated, early, and often self-limiting presentations at low disease prevalence, in patients with several interacting conditions and complex medication regimens. Dominant benchmarks are not built around that. HealthBench, the most prominent recent effort, is a genuine advance on multiple-choice testing: 5,000 multi-turn conversations scored against rubrics written by 262 physicians [18]. But each conversation is self-contained, and the contexts it names are emergencies, transforming clinical data, and global health rather than continuing generalist care. Nothing in it measures performance across repeated encounters with the same patient, the low-prevalence undifferentiated case-mix, or the accumulation of a longitudinal record. A systematic review of the testing and evaluation of health-care applications of LLMs found the same gap system-wide: of 519 studies, only 5% used real patient-care data, 95% used accuracy as the primary dimension of evaluation, and barely 1% measured calibration or uncertainty [19]. Existing evaluations test knowledge recall, single-encounter reasoning, or record-level language tasks rather than continuity [20–22], and we are aware of no benchmark that adequately simulates multimorbidity, longitudinal reasoning, or continuity-based decision-making. Conventional accuracy metrics also overlook what primary care turns on: longitudinal risk recognition, shared decision-making, and the slow evolution of symptoms. The empirical base is correspondingly thin: a scoping review of randomised controlled trials of AI in clinical practice found them concentrated in gastroenterology, radiology, surgery, and cardiology, with markedly less attention to primary care than to specialty care [23]. Polypharmacy and complex drug-drug, drug-disease, and disease-disease interactions were never solved by older algorithmic approaches and frequently exceed the capacity of individual GPs as well. This is where the next GenAI challenge in primary care lies, and precisely what current evaluation does not measure.

### Relational

Primary care is delivered across a relationship that persists over years. Current models are designed for isolated tasks and carry no persistent memory of the individual patient’s context from one encounter to the next, and were never grounded in that context to begin with. We call this contextual amnesia deliberately: not a claim that the model forgets knowledge it once held, but that the patient-specific context is absent by construction. Where continuity is paramount this matters as much as raw accuracy, and the value of continuity is well documented in the Nordic evidence base, where longer GP-patient relationships are associated with lower use of out-of-hours care, fewer acute admissions, and lower mortality in a dose-response pattern [24]. A second relational harm is subtler. GenAI tools used for documentation may paraphrase or normalise patient language, eroding the semantic precision on which diagnosis, legal clarity, and ethical care depend [25,26]; in mental health, informed consent, and narrative-medicine contexts, a shift in the patient’s own words can itself distort clinical meaning. The gap is narrowing as architectural responses improve, but it has not closed.

### Organisational

A first obstacle precedes deployment: primary-care voices are largely absent from the teams that build these systems, so the workflow assumptions are fixed before any GP sees the tool. GenAI has then been introduced into practices without adequate attention to how healthcare teams actually work, usually as an additional tool, comparable to a new ultrasound machine that merely has more functions and is more modern. The initial effort should have gone into training staff to integrate the new approach into everyday tasks rather than into deployment alone. Rather than augmenting GP roles, poorly integrated tools risk disrupting established practices and professional responsibilities. Deployment also presupposes robust digital infrastructure that is not available in all primary-care settings, particularly under-resourced or rural ones; this public-infrastructure mismatch may widen existing disparities in access and quality.

### Cultural

Population-level inequities are embedded in model training. Ethnolinguistic minorities, migrants, and socio-economically disadvantaged groups remain underrepresented in LLM corpora, producing impaired generalisability and culturally insensitive outputs [13,27]. Widely used models are predominantly built on Western values [28], and whether they handle differing forms of politeness or culturally sensitive terms adequately is unclear. Initiatives such as CultureLLM [29] and L2M3 [30] address this gap but have not been systematically tested for cultural sensitivity in medical contexts, and a comprehensive assessment of racist or ethnocentric bias is still lacking. This is not a problem experienced uniformly across settings. Primary care carries the most heterogeneous patient population in the health system, encounters it without pre-selection, and is where cultural misreading translates directly into missed presentations and unequal access. Any LLM intended for primary care should therefore be evaluated for cultural sensitivity and for performance across the specific populations the setting actually serves, as a condition of deployment rather than as later research.

### Linguistic

The Nordic countries and Europe more widely run primary care in languages that are poorly represented in training corpora relative to English, and primary care is the part of the system where consultations are least likely to be conducted in a shared first language and most likely to depend on register, idiom, and health literacy rather than on formal clinical vocabulary. Performance demonstrated in English on structured clinical text is not evidence of performance in Swedish, Danish, Norwegian, Finnish, or Icelandic on spoken, colloquial, interpreter-mediated consultation material, still less on the languages of migrant populations within those countries. Language is therefore an evaluation requirement in its own right and not a localisation step to be handled after validation.

### Epistemic

The epistemic foundations of GenAI, pattern recognition and language prediction, do not align with the reasoning clinical decision-making requires, and can produce outputs that are linguistically coherent yet clinically invalid. Even where GPT-4 has imitated clinical reasoning, it does not reason as a clinician does, which is why models must generate a rationale clinicians can inspect and validate [31]. Hallucination is the sharpest instance, and it is a general property of current LLMs rather than a primary-care problem as such: faced with knowledge gaps, models confidently fabricate trial results, guidelines, or drug interactions, because token prediction is probabilistic [32,33]. What makes it particularly consequential in primary care is the setting. Diagnostic uncertainty is the normal state, so a fabricated finding cannot be checked against a confirmed working diagnosis. Base rates are low and presentations undifferentiated, so a plausible but false suggestion is harder to exclude on prior grounds than in a triaged hospital population. Errors enter a record that is read for years, by other clinicians and across sectors, so a single fabrication acquires longitudinal reach. And because much of primary care rests on relational knowledge that is never written down, an output contradicting what the GP knows about the patient is detectable, whereas one filling a gap the GP cannot check is not. Fine-tuning and instruction alignment reduce the frequency of hallucination but do not eliminate it, and offer no deterministic guarantee that a harmful error will not recur; a deployment resting on good-enough accuracy is therefore misplaced, because the stochastic core of an LLM cannot meet frontline reliability thresholds without verifiable anchors. The consequence in primary care is also medico-legal: the GP signs the record, and fabricated content in it exposes the practitioner to liability. Evidence on use in interaction points the same way: LLMs do not significantly improve clinicians’ diagnostic reasoning compared with conventional resources on cases derived from real patients, although the models alone sometimes outperform physicians, probably reflecting sensitivity to prompt formulation [34]; and in a randomised preregistered study, members of the public assisted by LLMs identified conditions and dispositions no better than controls, despite the same models performing near-ceiling when tested alone [35]. Capability measured in isolation does not predict performance in interaction, which is the only mode in which primary care uses these tools.

These limitations, weak longitudinal reasoning, no built-in verification, difficulty across complex journeys, are what the emerging generation of agentic systems aims to address. Rather than a single model, agentic designs orchestrate several cooperating components: one recent example pairs a conversational agent with a separate module that continuously monitors it against safe clinical boundaries [36]. This is a genuine advance, and its core move, one component externally checking another, is the principle of decoupled verification we argue for below. But it relocates the governance problem rather than dissolving it: the monitoring component is itself supplied and trained by the vendor, so the question of who verifies the verifier moves up one level, and independent assurance remains necessary. Conversational diagnostic systems have themselves been evaluated in the peer-reviewed literature [37], but this decoupled-monitor architecture has not: the most prominent description of it is at present a corporate technical blog post, which we treat as contextual rather than primary evidence [36].

## A European and Nordic research and implementation agenda

European and Nordic primary care is not homogeneous: systems differ in gatekeeping, list structures, financing, organisation, and language, and any agenda must accommodate that diversity rather than assume a single model. Addressing the six misalignments above requires a coordinated agenda rooted in three principles: transparency, equity, and contextualisation.

**Transparency** entails not only disclosing model provenance and architecture, but also clarifying how outputs are generated, audited, and updated. Given the opaque data origins of current frontier systems, transparency is foundational both to clinical trust and to regulatory scrutiny.

**Equity** demands that models be trained and validated on data representative of Europe’s diverse linguistic, socio-economic, and clinical realities [13,27]. Current systems disproportionately reflect Anglophone, hospital-based datasets, underrepresenting rural, marginalised, and multimorbid populations [13,27]. How equity is to be addressed in practice can be stated operationally: evaluation datasets should be stratified in advance by language, migration background, age, multimorbidity count, and area-level deprivation; performance should be reported per stratum rather than in aggregate; a maximum acceptable performance gap between strata should be declared before testing rather than after; and a model that widens an existing care gap should fail assessment on that ground alone. The Nordic countries, with their register infrastructure, national patient identifiers, and equity-driven primary care systems, are unusually well placed to supply the stratified data this requires.

**Contextualisation** requires integration into real-world workflows through a primary-care-led validation sandbox, anchored in the European Health Data Space (EHDS) [38] and operationalised through existing national data-governance arrangements. EU members (Sweden, Denmark, Finland) participate directly in the EHDS Regulation, while Norway and Iceland will need integration through their EEA framework agreements; this is solvable, but it cannot be ignored if the agenda is to be genuinely Nordic-European rather than EU-only. Grounding can be built at two levels. Regionally, linked primary-care registers and national patient identifiers allow models to be calibrated and evaluated on the actual case-mix, referral patterns, and language of a given health system rather than on imported hospital data. At practice level, structured record data, longitudinal episodes, primary-care coding, and medication histories can anchor retrieval and verification to the individual patient’s record under local governance. Neither requires a single pan-Nordic dataset; both are achievable through collaboration between the Nordic colleges and their national frameworks. Without such infrastructure, GenAI risks becoming a technological appendage rather than a healthcare asset.

### Organising and funding independent verification

Independent assurance is easy to demand and hard to build, so we state how we think it should be organised, paid for, and judged.

#### Organisation

No new agency is required. The function should be hosted by bodies that already carry the mandate: the Nordic medical-products agencies (the Swedish Läkemedelsverket, the Danish Lægemiddelstyrelsen, the Norwegian Direktoratet for medisinske produkter (DMP, Norwegian Medical Products Agency), the Finnish Fimea, and the Icelandic Lyfjastofnun), notified bodies designated under the EU Medical Device Regulation, and primary-care research networks supplying the case material, the clinical sign-off, and the practice sites for prospective testing. This distributes the work as regional assurance nodes rather than one central laboratory, keeping evaluation close to the language and case-mix being evaluated. The substance is that proposed for the network of health AI assurance laboratories in the United States [39]: dataset audits, adversarial hallucination stress tests, and continuous performance monitoring at scale, gated by an independent body rather than the model vendor.

#### Funding

Three routes are realistic, and they are not mutually exclusive. Market-entry and model-update fees paid by the vendor, on the notified-body precedent, are the most immediately available, but only if the fee is pooled and the assessor is assigned rather than chosen and paid directly by the party being assessed. Public funding as health-system infrastructure, in the same budget line as pharmacovigilance and register maintenance, is appropriate for the post-deployment monitoring component, which no vendor has an incentive to fund. EU and EEA co-funding through EHDS implementation is the natural route for shared evaluation datasets and tooling. The design constraint that matters more than the source is that the assessor’s income must not depend on the assessed party’s satisfaction.

#### Criteria

Effectiveness and safety should be judged on: performance on primary-care case-mix rather than hospital benchmarks, reported per stratum as above; the proportion of clinical outputs traceable to a resolvable source, and the rate of unsupported assertions under adversarial stress testing, against thresholds declared in advance; behaviour under model update, with re-assessment triggered by any substantive version change rather than by calendar; human-factors testing with real users in real workflows rather than simulation alone, given how sharply interactive performance diverges from standalone performance [35]; and post-deployment surveillance of incident reports, performance drift, and clinician override rates. Results, including failures, should be public.

Finally, all GenAI strategies should align with the EU AI Act [40], which mandates transparency, risk classification, and sector-specific governance. AI systems that are medical devices required to undergo third-party conformity assessment under the Medical Device Regulation are classified as high risk under Article 6(1), whose corresponding obligations apply from 2 August 2027. For the Nordic non-EU context, equivalent obligations under the EEA Agreement and national regulation provide a parallel basis.

## Strategic recommendations and success criteria

Responsible deployment requires a shift from general-purpose black-box commercial models toward transparent, medically governed systems: models trained on representative, longitudinal, community-based datasets curated through public infrastructures such as the EHDS [38], subject to continuous algorithmic audit, and equipped with interpretability protocols that let clinicians understand, challenge, or override outputs [8,41]. Deterministic knowledge assets should be combined with generative components: retrieval-augmented generation (RAG) pipelines require every response to reference current guidelines or structured electronic health record (EHR) excerpts, constraining conjecture and enabling post-hoc verification [42].

GenAI should be embedded within team-based care as a tool, not as a participant. We are explicit about this because the language of team integration invites the opposite reading: these systems are instruments operated by an identified clinician who remains accountable for the output, they hold no role, no scope of practice, and no accountability of their own, and no part of the care team’s responsibility transfers to them. Within that constraint, the **Five Rights framework** for Clinical Decision Support offers a useful guide [43]: (1) the **right information**, evidence-based and patient-specific, is delivered to (2) the **right person**, the appropriate accountable member of the multi-disciplinary team; (3) in the **right format**, clear and concise; (4) through the **right channel**, integrated directly into the EHR for interoperability; and (5) at the **right time**, the precise moment it is needed within the clinical workflow. Because agentic architectures place one model in front of another, the framework must be read for multi-agent settings and not for single tools: the accountable person is a human being at the end of the chain, not the supervising model.

Patient-facing use requires its own safeguards, and the risk here is specific. If an LLM collects patient-reported history before or between consultations, the model’s summary is a generated text, not the patient’s account, and it may omit, normalise, or invent. Three conditions follow. Patient-reported material should enter the record flagged as patient-attributed and unverified, kept separate from clinician-authored content and never silently merged into it. The patient’s own wording should be retained alongside any model summary so that the summary can be checked against the source rather than replacing it. And no model-generated content should become a clinical statement in the record without explicit clinician verification, logged as such. Patients should be told when a system is in use, what happens to what they enter, and how to decline it without disadvantage. Without these conditions, patient involvement becomes an unmonitored route for fabricated content to enter a longitudinal record, which is the failure mode the rest of this paper argues against.

Figure 1 places these elements in relation to one another as a layered architecture: the patient relationship at the top, and beneath it the GP-led team, the tool itself, independent assurance, the data infrastructure, and the regulatory ground. Trust reaches the top only if every layer below it holds. Table 1 summarises the same roadmap and, for each recommendation, states the criterion by which success should be judged. The criteria are deliberately measurable, and several of them are measurable specifically because Nordic register infrastructure makes them so. Taken together, they define what successful implementation would mean: no measurable increase in diagnostic error or delayed diagnosis against baseline; documentation burden reduced without loss of record accuracy; every clinical output traceable to a source; no widening of performance gaps between patient groups; continuity and clinician override preserved rather than eroded; and independent re-assessment at every substantive model update. A programme that cannot show these is not a successful implementation regardless of benchmark scores.

**Figure 1.**
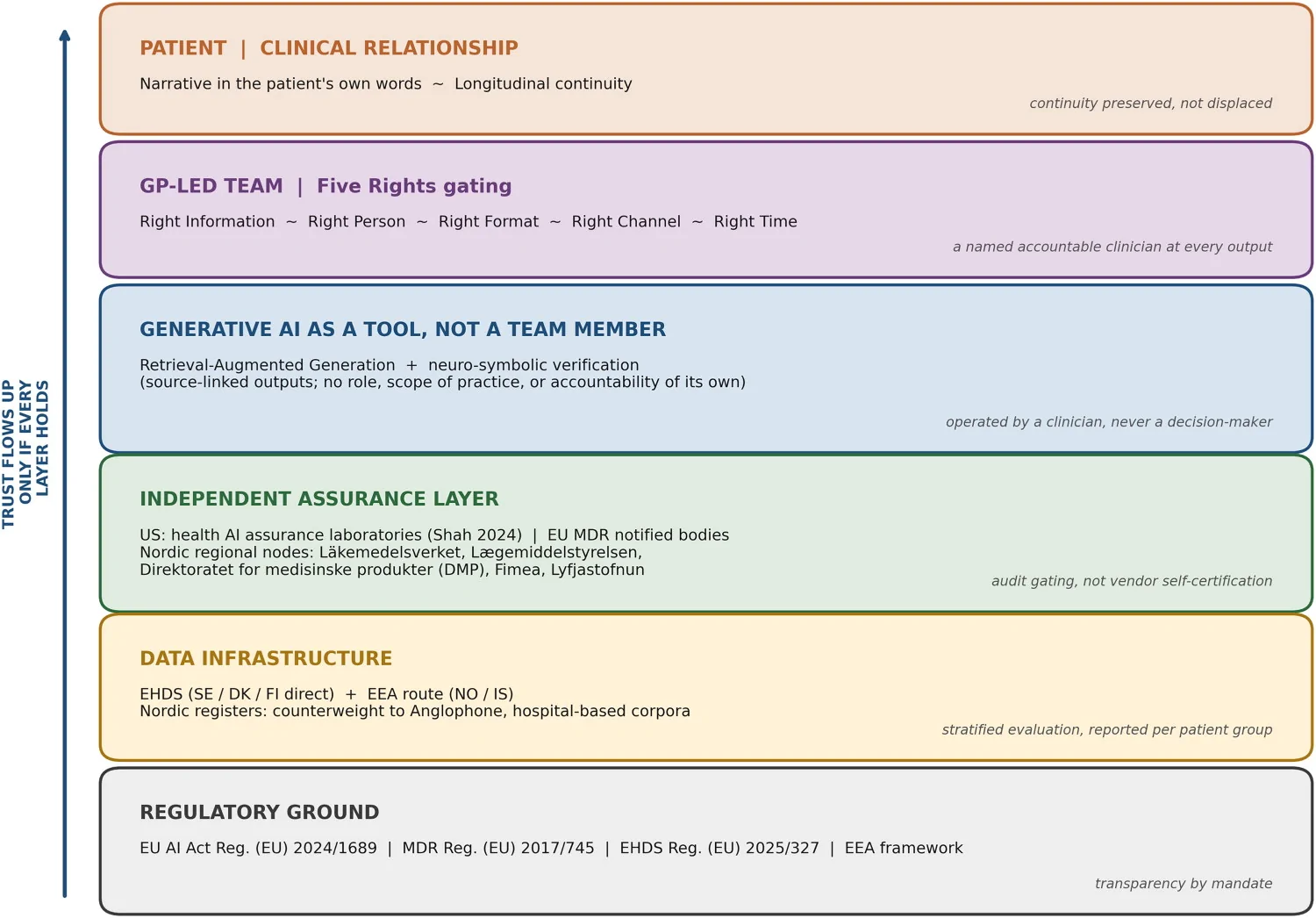
The Blueprint of Trust: a six-layer governance architecture for generative AI in primary care. Trust does not arise from model accuracy alone; it accrues only when each layer holds for the patient-GP relationship at the top. The generative model sits in the stack as a tool operated under clinician accountability, not as a member of the care team.

**Table 1.** Strategies for implementing GenAI to create a blueprint of trust in primary care.

Strategic category	Key recommendation	Goal / outcome	Success criterion: how we would know
System architecture	Shift from black-box models to open-source, domain-specific LLMs trained on community-based datasets (e.g. EHDS)	Adaptability, no proprietary lock-in, fit for primary care	Model provenance, training-data description, and update history published; independent re-evaluation possible without vendor cooperation
Safety & verification	Implement RAG and neuro-symbolic stacks linking LLMs to current guidelines and EHR data	Constrain conjecture, make every factual claim checkable, provide inspectable reasoning	Proportion of clinical outputs with a resolvable source citation approaches 100%; unsupported-assertion rate under adversarial stress testing below a pre-declared threshold
Accountability	Establish independent assurance nodes, hosted by medical-products agencies, notified bodies, and primary-care research networks, for stress tests, audits, and continuous monitoring	Move trust from charismatic models to quantifiable proof of safety	Assessment before market entry and at every substantive model update; results, including failures, published; assessor not paid directly by the assessed vendor
Clinical operation	Apply the Five Rights framework: Right Information, Right Person, Right Format, Right Channel (EHR integration), Right Time	Support the care team rather than adding tasks	Documentation time per consultation falls with no loss of record completeness or accuracy on audit; no increase in alert or task volume per clinician
Role in the team	Deploy GenAI as a tool operated by a named accountable clinician, never as a team member with a role of its own	Human accountability preserved; roles and workflows reconfigured around the tool, not delegated to it	Every clinical output attributable to an accountable human; override remains available and its use is monitored, not suppressed
Patient involvement	Use LLMs to capture patient-reported history under safeguards: patient-attributed and unverified by default, original wording retained, clinician verification logged before anything becomes a clinical statement	Add value to the encounter without letting unverified or fabricated content enter the record	Zero unverified model-generated content in the clinical record on audit; patients informed and able to decline without disadvantage
Equity & language	Evaluate systematically for cultural sensitivity and for performance across the heterogeneous populations primary care actually serves, in the languages of consultation	Equitable performance rather than aggregate performance	Results reported per stratum (language, migration background, age, multimorbidity, deprivation); gap between strata within a pre-declared margin; no widening of an existing care gap
Policy & ethics	Align with the EU AI Act and, for EEA Nordic countries, equivalent national obligations	Governance and primary-care-led validation	Documented risk classification and conformity route per deployed system; primary-care representation in the validating body
Continuity of care	Protect the relational and longitudinal core of primary care as an explicit design constraint	Technology augments the care relationship rather than displacing it	No reduction in continuity indices or in patient-reported relational measures after deployment

### What is specific to primary care

Some of what we recommend is general good practice for clinical AI: source-linked outputs, independent assurance, published assessment results, alignment with the AI Act. We claim no originality for these and include them because a primary-care agenda that omitted them would be incomplete.

Four of our recommendations are specific to primary care and would not be generated by a hospital-facing agenda. First, benchmarks constructed around low prevalence, undifferentiated presentation, and multimorbidity, because that case-mix is the defining feature of the setting and is absent from existing evaluation. Second, longitudinal and continuity-preserving evaluation, because the unit of care in primary care is a relationship over years rather than an episode, and no current benchmark measures performance across repeated encounters with the same patient. Third, evaluation in the languages and registers of actual consultation, including interpreter-mediated and low-health-literacy communication, because primary care is where unselected linguistic diversity arrives. Fourth, the patient-attributed-and-unverified handling of patient-reported material, because primary care is the setting in which patients supply most of the history and in which the record is longitudinal enough for a single fabrication to persist. These four are where a primary-care-led agenda changes what gets built, rather than restating what is already known about LLMs.

## Conclusions

GenAI will not earn trust in primary care through accuracy alone. That models can match physicians on case-based reasoning makes the point sharper, not weaker: capability measured in hospital settings does not transfer automatically to the longitudinal, relational, multimorbid work of primary care, and some uses currently proposed may not survive evaluation under primary-care conditions at all. What is required instead is the architecture set out here: benchmarks built for primary care; data that reflect its real populations and languages; outputs tied to verifiable sources and assessed independently; deployment as a tool under the Five Rights with human accountability intact; governance extending to multi-agent systems; and success criteria fixed in advance, so that implementation can be judged rather than asserted. Nordic primary care, with its register-driven research tradition, has the empirical foundation to lead this work rather than follow imported templates.

## Use of generative artificial intelligence

During the preparation of this manuscript the authors used Anthropic Claude for drafting and restructuring text, for language editing, for proposing candidate sources, and for preparing the point-by-point response to the reviewers. Generative AI was not used to generate, analyse, or interpret data, and it did not determine the analysis or the recommendations, which are the authors’ own. Every source proposed with such assistance was subsequently checked by the authors against the primary record, and citations that could not be confirmed were removed. The authors reviewed and edited all resulting text and take full responsibility for the content of this article, including any part prepared with the assistance of generative AI. No generative AI tool is listed as an author.

## List of abbreviations

AI: Artificial Intelligence
DMP: Direktoratet for medisinske produkter (Norwegian Medical Products Agency)
EEA: European Economic Area
EHDS: European Health Data Space
EHR: Electronic Health Record
EU: European Union
GenAI: Generative Artificial Intelligence
GP: General Practitioner
LLM: Large Language Model
RAG: Retrieval-Augmented Generation.
